# Parametric Optimization of a Star-Shaped Bluff Body for Enhanced VIV-Galloping Coupled Energy Harvesting

**DOI:** 10.3390/mi17050616

**Published:** 2026-05-17

**Authors:** Li Zhang, Hai Wang, Chunlai Yang, Weiwei Duan, Jingjing Peng

**Affiliations:** 1 School of Mechanical Engineering, Anhui Institute of Information Technology, Xinwu Campus, Wuhu 241000, China; 17856929892@163.com (L.Z.); 15655357889@163.com (W.D.); 19955432023@163.com (J.P.); 2 College of Mechanical and Automotive Engineering, Anhui Polytechnic University, International Engineer College Campus, Wuhu 241000, China; ycl@ahpu.edu.cn; 3 Anhui Province Key Laboratory of Advanced Numerical Control & Servo Technology, Wuhu 241000, China

**Keywords:** VIV-galloping, star-shaped bluff body, wind-driven vibratory energy harvester

## Abstract

Under low wind speed conditions, conventional bluff body energy harvesters suffer from a single vibration mechanism and a narrow effective wind speed range, making it difficult to meet the continuous power supply demands of miniature electronic devices. In this paper, by systematically optimizing the number of triangular prisms *N* and the circumferential installation angle α, a parametrically adjustable star-shaped energy harvester (SEH) is proposed. The proposed structure consists of a cylindrical base with a tunable number of triangular prisms uniformly distributed along its circumference, aiming to reveal the regulation mechanism of the VIV-galloping coupling response and energy harvesting performance. Conceptual design and theoretical modeling of the SEH are first carried out. Then, three-dimensional fluid–structure interaction simulations are performed by varying *N* and *α*, and a prototype is fabricated for wind tunnel experimental validation. The results show that under the optimal parameter combination of *N* = 7 and *α* = 51.4°, the SEH achieves a maximum output voltage of 12.2 V at a wind speed of 3.41 m/s, with a maximum output power of 1.488 mW, and the effective wind speed range is broadened to 2.5~12.44 m/s. Compared with the conventional cylindrical energy harvester (CEH), the SEH (*N* = 7) increases the maximum output voltage by 44.38%, the maximum output power by 108.4%, and expands the effective wind speed range by 198.50%. Through systematic optimization of key geometric parameters, this study achieves synergistic regulation of flow-induced vibration modes and performance enhancement, providing a parametric design basis for efficient low-speed wind energy harvesting, which can promote the development of self-powered technologies for micro-sensors and IoT devices.

## 1. Introduction

The rapid advancement of technologies such as Wireless Sensor Networks and the Internet of Things has led to increasing challenges in power supply. Traditional batteries, constrained by their finite lifespans and risks of environmental pollution, struggle to meet the long-term power supply demands of distributed devices [1,2]. Ambient vibration energy harvesting technology, leveraging its inherent advantages of sustainable energy supply and maintenance-free operation, has emerged as a highly promising alternative to address this long-standing challenge [3,4,5]. Various ambient energy sources—including wind energy [6], acoustic energy [7], wave energy [8], and solar energy [9]—are harnessed for energy harvesting, with electrostatic [10], piezoelectric [11], electromagnetic [12], and triboelectric [13] effects widely applied in this process. Among these, wind-driven flow-induced vibration energy harvesting (FIVEH) has demonstrated significant application prospects in the micro-energy field due to its wide resource distribution and strong environmental adaptability [14,15,16].

Current research on FIVEH primarily focuses on vortex-induced vibration energy harvesters (VIVEH) [17] and galloping-based vibration energy harvesters (GVEH) [18]. Specifically, for VIVEH, the bluff body is typically a cylinder. When fluid flows around the bluff body, periodic vortex shedding occurs in its wake, which induces the bluff body into resonance. Its advantages lie in the stable amplitude and high energy conversion efficiency within the lock-in range [19]. To enhance the performance of VIVEH, researchers have employed several strategies, such as incorporating splitter plates [20], adopting grooved surface modifications [21], integrating metasurfaces [22], applying magnetic forces [23], and adopting twin cylinders [24]. Hu et al. [25] developed an optimized vortex-induced vibration (VIV) wind energy harvester by symmetrically attaching small-diameter cylindrical rods at circumferential positions of θ = 60° on both sides of a main cylinder, enabling continuous energy harvesting across a wider range of wind speeds. Naseer et al. [26] designed a VIV piezoelectric energy harvester incorporating nonlinear attractive magnetic forces. Their experimental studies demonstrated that by optimizing both the interaction spacing and the electrical load resistance, the harvester achieved efficient broadband energy harvesting. Jia et al. [27] proposed a wind energy harvester based on an asymmetric cylinder, which undergoes VIV-induced bending and torsional vibrations to harvest wind energy at low wind speeds. For GVEH, the bluff body typically adopts geometries such as a square or triangular prism with a specific angle of attack. When fluid flows past the structure, variations in the effective angle of attack trigger aerodynamic instability, which induces large-amplitude structural vibrations. This mechanism endows GVEH with the advantages of broadband frequency response and large-amplitude output [1]. To enhance galloping performance, researchers have employed strategies including bluff body shape modification [28,29] and nonlinear magnetic force integration [30,31]. Poude et al. [32] designed a galloping-based piezoelectric wind energy harvester with passive shape attachments. Experimental results demonstrated that its voltage and power output both exceeded those of comparative configurations. Wang et al. [33] designed a tristable galloping-based piezoelectric energy harvester using nonlinear magnetic forces. This tristable design reduced the threshold wind speed to 1.0 m/s, achieving an output power of 0.73 mW at 7.0 m/s.

Currently, researchers have attempted to optimize energy harvesting efficiency by coupling VIVEH with GVEH. Wang et al. [34] designed piezoelectric wind energy harvesters using bluff bodies with circular/square hybrid cross-sections, enhancing performance by optimizing cross-section combinations and angles of attack to couple VIV and galloping. Experimental and theoretical studies demonstrated that the performance was significantly improved compared to traditional VIV or galloping harvesters. Zhou et al. [35] developed a bistable wind energy harvester. Experimental results demonstrated that the device can effectively generate electricity at wind speeds exceeding 1.3 m/s. Tian et al. [36] designed a dumbbell-shaped piezoelectric energy harvester, whose experimental and simulation data confirmed the effectiveness of this design in exploiting VIV and galloping for enhanced performance. Huang et al. [37] designed a wind energy harvester using a D-shaped hybrid bluff body with a preset angle of attack. Experimental results demonstrate that the optimal configuration achieved an output of 38.76 V_RMS_ (Root Mean Square Voltage), representing a 153.5% improvement compared to conventional galloping-based harvesters. The above studies have well demonstrated the potential of the VIV-galloping coupling mechanism in broadening the effective wind speed range and enhancing output performance.

However, most of these works adopt single or only a few geometric configurations, focusing on validating the effectiveness of the coupling effect, while lacking systematic parametric optimization on key geometric parameters that affect coupling intensity, such as the number of attached prisms and their circumferential distribution angle. Notably, recent studies have further demonstrated that dynamic regulation and parametric optimization are effective approaches to enhance energy harvesting performance. For instance, Zhao et al. [38] achieved a 46.91% increase in average power for a wearable biomechanical energy harvester through a self-aligning mechanism. Chen et al. [39] proposed an adaptive underwater biomechanical energy harvesting belt (AU-BEHB) with a self-regulating excitation angle and a flexible adaptive excitation path, attaining average powers of 2.09 W and 2.29 W under 2 Hz and 300 mm excitation. Zhao et al. [40] introduced nonlinear magnetic force, variable stiffness, and adaptive-anastomotic barricades in rotational energy harvesting, achieving average power outputs of 1600 μW for the piezoelectric unit and 205.1 μW for the triboelectric unit over a wide rotational speed range of 0–1000 r/min. These studies confirm, from different perspectives, the crucial role of dynamic regulation/adaptive design in broadening the operating range and enhancing output power.

To this end, this paper proposes a parametrically adjustable star-shaped bluff body structure. It consists of a cylindrical base with a tunable number of triangular prisms uniformly distributed along its circumference. The number of prisms *N* and the circumferential installation angle α are adopted as design variables. Through a systematic parametric optimization strategy, this study aims to reveal the influence of these two parameters on the VIV-galloping coupling performance, thereby obtaining the optimal parameter combination and achieving broadband high-efficiency energy harvesting. In terms of performance evaluation, the output power is calculated from the output voltage under a fixed load resistance, and a quantitative power-level comparison is performed between the SEH and the conventional cylindrical energy harvester (CEH) to fully demonstrate the practical contribution of parametric optimization to energy harvesting performance.

The remainder of this paper is organized as follows. Section 2 presents the design concept of the proposed star-shaped energy harvester (SEH). Section 3 provides the mathematical modeling of the SEH. Section 4 describes the computational fluid dynamics (CFD) simulations. Section 5 introduces the experimental setup. Section 6 reports the experimental validation results along with the parameter study. Finally, Section 7 concludes this study.

## 2. Energy Harvester Design

**Figure 1:**
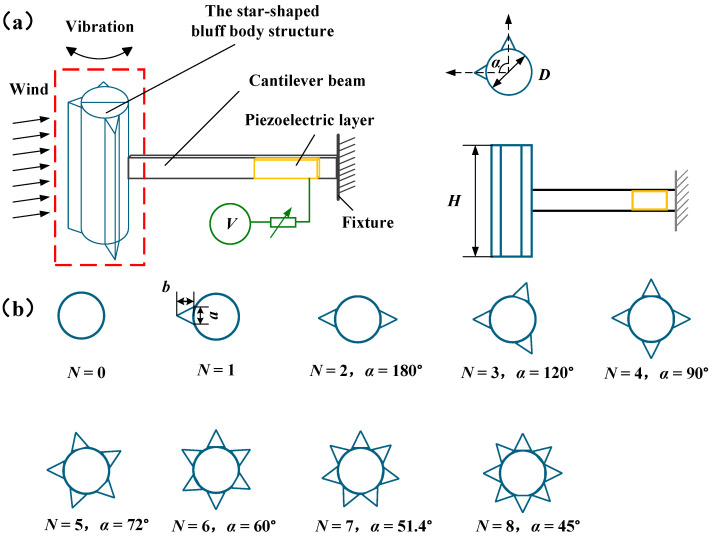
Schematic diagram of the SEH: (**a**) Overall configuration; (**b**) Detailed geometry of the star-shaped bluff body.

To this end, this paper presents a parametrically adjustable SEH, with its prototype shown in Figure 1. The SEH consists of a cantilever beam, piezoelectric patches, and a star-shaped bluff body. It adopts a horizontal cantilever configuration (beam axis parallel to the flow direction, as shown in Figure 1a) rather than a vertical one (axis perpendicular to the flow direction). This is because the horizontal cantilever offers lower bending stiffness in the transverse direction (perpendicular to the flow), facilitating large-amplitude transverse oscillations of the bluff body to excite VIV-galloping coupling. Furthermore, the horizontal arrangement avoids beam intrusion into the wake region that could disturb vortex street formation, and the strain concentration at the root is more favorable for piezoelectric energy conversion. When the SEH is immersed in an airflow with the direction fixed along its axis (i.e., 0° angle of attack), the star-shaped bluff body induces vibration, driving the cantilever beam to bend. Consequently, the piezoelectric patch attached to the fixed end of the beam undergoes mechanical deformation, thereby generating electrical charge. Figure 1b details the geometric design of the star-shaped bluff body: a cylindrical base with multiple triangular prisms uniformly distributed along its circumference as adjustable structural variables, while the dimensions of the prisms remain fixed. The shape of the star-shaped bluff body is governed by two key parameters—the circumferential installation angle α and the number of triangular prisms *N*. Here, *α* is defined as the angle between the longitudinal centerlines of two adjacent triangular prisms and takes values of 45°, 51.4°, 60°, 72°, 90°, 120°, and 180° in this study. The number of triangular prisms *N* is set to 0, 1, 2, 3, 4, 5, 6, 7, and 8. For reference, *N* = 0 in Figure 1b corresponds to the Cylindrical bluff body Energy Harvester (CEH).

## 3. Mathematical Model

The SEH is modeled as a single-degree-of-freedom system composed of a spring, a mass, and a damper [41]. Herein, the lumped parameter method [42,43] is employed to couple the dynamic equation of the system with the piezoelectric constitutive equations via a coupling coefficient.

(1) $$m\ddot{x}(t)+c\dot{x}(t)+kx(t)-\Theta V(t)=F_{a}(t)$$

Here, *m* represents the equivalent mass of the system; c denotes the equivalent damping; *k* stands for the equivalent stiffness; Θ is the electromechanical coupling coefficient; *V*(*t*) refers to the output voltage of the system; and *F*_a_(*t*) represents the force induced by vortex-induced vibration and galloping.

According to the quasi-steady theory, the aerodynamic force *F*_a_(*t*) is given by [44].

(2) $$F_{a}(t)=\frac{1}{2}\rho U^{2}DH(C_{L}+C_{D}\tan\beta )\sec\beta$$

In this formulation, *ρ* is the fluid density; *U* is the fluid velocity; *D* is the length of the windward face; *H* is the height of the windward face; *C_L_* and *C_D_* are the lift and drag coefficients, respectively, determined via experiments or CFD simulations; and *β* is the angle of attack, $\beta =\arctan\frac{\dot{x}(t)}{U}$.

Substituting Equation (1) into Equation (2), we obtain

(3) $$m\ddot{x}(t)+c\dot{x}(t)+kx(t)-\Theta V(t)=\frac{1}{2}\rho U^{2}DH(C_{L}+C_{D}\tan\beta )\sec\beta$$

When the system is connected to an external circuit, the current equation, according to Kirchhoff’s current law, is given by

(4) $$\frac{V(t)}{R_{L}}+C_{p}\dot{V}(t)+\Theta \dot{x}(t)=0$$

where *R_L_* is the load resistance and *C_p_* is the equivalent capacitance of the piezoelectric patch, which is given by [45]:

(5) $$C_{p}=\frac{{\bar{\varepsilon }}_{33}^{s}bL_{b}}{h_{p}}$$

where ${\bar{\varepsilon }}_{33}^{s}$ is the dielectric permittivity component at constant strain; *b* and *L_b_* are the width and length of the cantilever beam, respectively; and *h_p_* is the thickness of the piezoelectric patch.

In summary, the coupled electromechanical equations for the composite system are given by

(6) $$\left\{\begin{array}{c} m\ddot{x}(t)+c\dot{x}(t)+kx(t)-\Theta V(t)=\frac{1}{2}\rho U^{2}DH(C_{L}+C_{D}\tan\beta )\sec\beta \\ \frac{V(t)}{R_{L}}+C_{p}\dot{V}(t)+\Theta \dot{x}(t)=0 \end{array}\right.$$

Substituting $\tan\beta =\frac{\dot{x}(t)}{U}$ and $\sec\beta =\sqrt{1+{\left(\frac{\dot{x}(t)}{U}\right)}^{2}}$ into Equation (2), the force term becomes

(7) $$F_{a}(\dot{x})=\frac{1}{2}\rho UDH\sqrt{U^{2}+{\dot{x}}^{2}}\left(C_{L}+C_{D}\frac{\dot{x}}{U}\right)$$

Thus, the governing electromechanical coupling equations for the SEH are rewritten as

(8) $$\left\{\begin{array}{c} m\ddot{x}(t)+c\dot{x}(t)+kx(t)-\Theta V(t)=F_{a}(\dot{x})\\ \frac{V(t)}{R_{L}}+C_{p}\dot{V}(t)+\Theta \dot{x}(t)=0 \end{array}\right.$$

Due to the nonlinearity introduced by $\beta =\arctan\frac{\dot{x}(t)}{U}$, the above system of equations is converted into a set of first-order ordinary differential equations. The state variables are defined as

(9) $$\left\{\begin{array}{c} y_{1}=x(t)\\ y_{2}=\dot{x}(t)\\ y_{3}=V(t) \end{array}\right.$$

Substituting Equation (9) into Equation (8) leads to the first-order system of ODEs:

(10) $$\left\{\begin{array}{c} {\dot{y}}_{1}=y_{2}\\ {\dot{y}}_{2}=\frac{1}{m}[F(y_{2})-cy_{2}-ky_{1}+\Theta y_{3}]\\ {\dot{y}}_{3}=-\frac{1}{C_{p}}\left(\frac{y_{3}}{R_{L}}+\Theta y_{2}\right) \end{array}\right.$$

## 4. CFD Simulation

**Figure 2:**
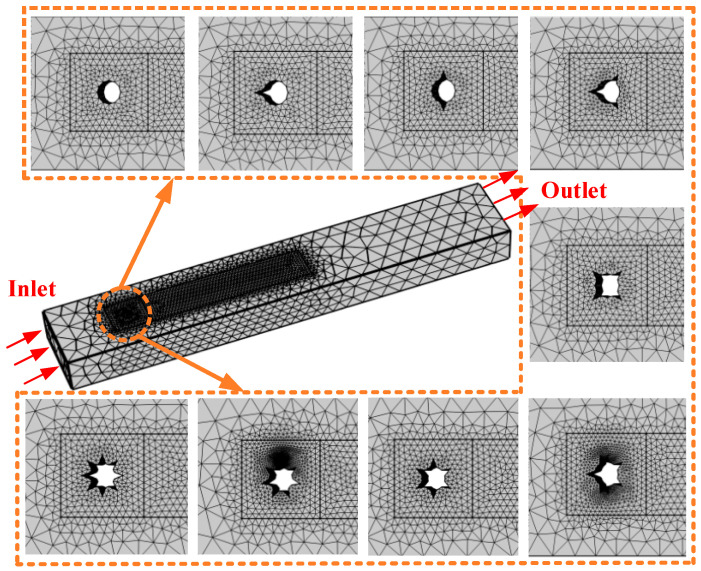
SEH (*N* = *i* (1 ≤ *i* ≤ 8)) and CEH (*N* = 0) mesh generation.

Figure 2 illustrates the graded meshing strategy for the SHE (*N* = *i* (1 ≤ *i* ≤ 8)) and the CEH (*N* = 0). The computational domain is a rectangular cuboid of size 39*D* × 6.40*D* × 3.75*D*, where *D* = 32 mm is the characteristic diameter of the cylindrical bluff body. The inlet is placed 6.25*D* upstream of the cylinder center, and the outlet is located 31.25*D* downstream to ensure full wake development. The inlet boundary is set as a velocity inlet, the outlet as a pressure outlet, and the bluff body surface as a no-slip wall. Turbulence is modeled using the SST *k*–*ω* model. Unstructured free tetrahedral meshes are adopted as the base mesh type to accommodate the geometric complexity. Based on the physical features of flow-induced vibration, the computational domain is divided into a core flow-induced region and a non-flow-induced region, with different mesh densities applied accordingly. The core region uses a refined mesh with element sizes in the range [1.66 mm, 8.22 mm] to capture vortex shedding, while the outer region uses a coarser mesh with element sizes in [11.7 mm, 54.9 mm] to reduce computational cost. Four boundary-layer mesh layers are set near the wall with a stretching factor of 1.2 to accurately resolve the near-wall flow. The surrounding close-up views in the figure show the local mesh details around different bluff body configurations, demonstrating the mesh’s adaptability to geometric features. A transient solver is employed with output time steps set as range (0, 0.2, 3.4) and range (3.5, 0.02, 7) (unit: s).

**Figure 3:**
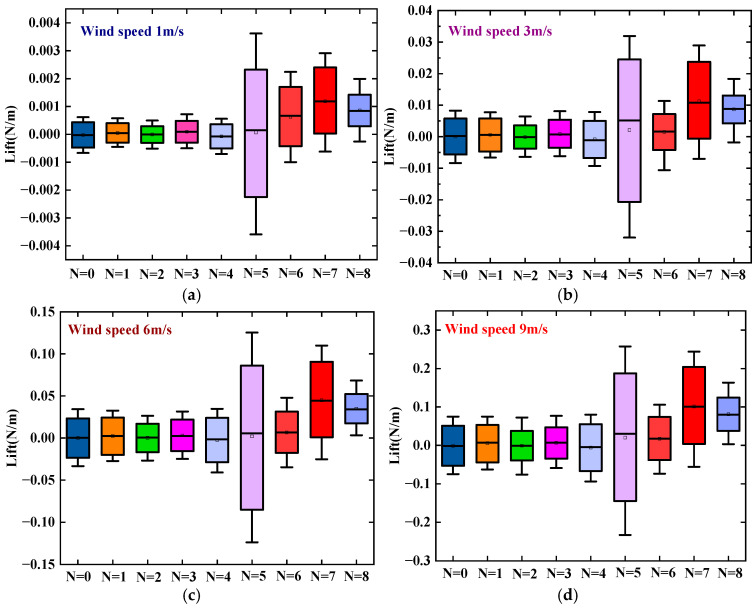
Lift of the SEH (*N* = *i* (1 ≤ *i* ≤ 8)) and CEH (*N* = 0) at different wind speeds; (**a**) 1 m/s; (**b**) 3 m/s; (**c**) 6 m/s; (**d**) 9 m/s.

Figure 3 presents the variation in lift force with wind speed for the SEH (*N* = *i* (1 ≤ *i* ≤ 8)) and the CEH (*N* = 0). As the wind speed increases from 1 m/s to 9 m/s, the lift amplitude of both the CEH and all SEH configurations increases exponentially. This is because the higher wind speed raises the vortex shedding frequency and the rate of change in fluid momentum per unit time, thereby amplifying the periodic excitation force acting on the bluff body. Meanwhile, at a given wind speed, the number of triangular prisms *N* has a significant effect on the lift amplitude. Notably, when *N* ≤ 4, the lift amplitudes of these configurations show little difference from that of the CEH (*N* = 0). This is because the low-density prism arrangement causes only a weak geometric perturbation to the flow field around the cylindrical base. Although the flow separation points are locally fixed by the prism edges, the mode and intensity of the wake vortex street have not fundamentally changed; consequently, the lift characteristics have not undergone a qualitative transition. When *N* ≥ 5, a clear jump in lift amplitude occurs. Among these, the SEH with *N* = 5 exhibits the largest fluctuation range of lift. The main reason is that five prisms already form a sufficiently dense asymmetric multi-edge layout around the circumference, which forces flow separation at each prism edge and generates a stronger and more irregular vortex street in the wake, thereby greatly enhancing the aerodynamic excitation force. However, the SEH with *N* = 7 achieves a maximum lift of 0.244 N/m at 9 m/s, which is 225% higher than that of the CEH (0.075 N/m) and 31% higher than that of the *N* = 5 configuration. When the number of prisms is further increased to *N* = 8, the lift shows a slight decrease (0.202 N/m at 9 m/s). This occurs because eight prisms are distributed almost continuously in the circumferential direction, making the outer contour of the bluff body approach a nearly circular polygon. As a result, the flow separation points are less sharply defined, and the aerodynamic instability tends to revert toward that of a smooth cylinder.

**Figure 4:**
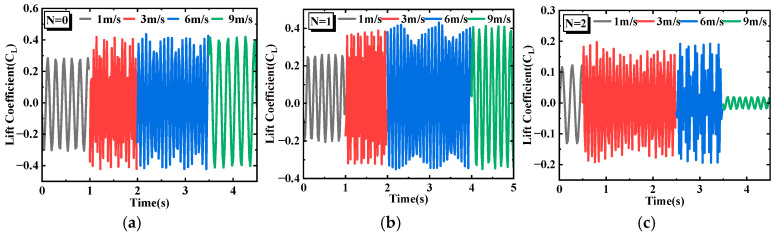

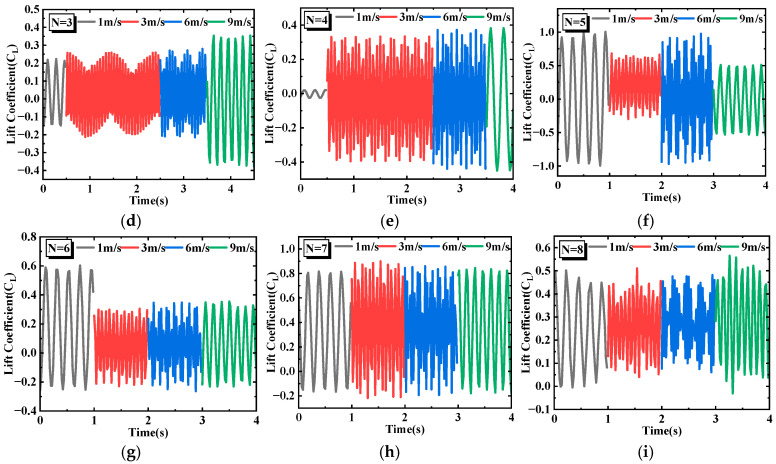
Time-history curves of lift coefficients for the SEH and CEH at different wind speeds. (**a**) *N* = 0 (**b**) *N* = 1 (**c**) *N* = 2 (**d**) *N* = 3 (**e**) *N* = 4 (**f**) *N* = 5 (**g**) *N* = 6 (**h**) *N* = 7 (**i**) *N* = 8.

Figure 4 shows the time-history curves of the lift coefficient for the SEH and CEH under different wind speeds. Generally, the lift coefficients of both the SEH and CEH exhibit nearly sinusoidal periodic fluctuations, but the amplitude and waveform morphology depend on the number of triangular prisms *N*. In Figure 4a, the CEH (*N* = 0) has the smallest fluctuation amplitude at 1 m/s. Between 3 m/s and 6 m/s, the amplitude suddenly increases and reaches a peak. Figure 4b shows that the time-domain lift coefficient of the SEH (*N* = 1) is essentially similar to that of the CEH, indicating that a single prism causes only a weak disturbance to the flow field around the cylinder and does not alter the dominant VIV mechanism. In Figure 4c (SEH, *N* = 2), the lift coefficient first increases and then drops sharply at 9 m/s. At 6 m/s, large fluctuations and high-frequency disturbances appear, suggesting that two prisms begin to destroy the wake symmetry and induce multi-frequency vortex shedding components. In Figure 4d (SEH, *N* = 3), the lift coefficient increases with wind speed. At 3 m/s, a distinct beating phenomenon is observed, caused by a slight difference between the vortex shedding frequency and the structural natural frequency, resulting in a linear superposition of the two frequency components. In Figure 4e (SEH, *N* = 4), the lift coefficient amplitude does not consistently exceed that of the CEH (*N* = 0) at different wind speeds. This is because the four prisms are approximately uniformly distributed around the circumference, and their geometric perturbation “blunts” the cylindrical surface. In summary, when the number of prisms *N* ≤ 4, the geometric perturbation is weak. The flow field is still dominated by the cylindrical base, and the prisms cause only local flow separation, insufficient to fundamentally alter the mode or intensity of the wake vortex street. Consequently, the lift coefficient amplitude shows no significant difference from that of the CEH (*N* = 0).

In Figure 4f (SEH, *N* = 5), the lift coefficient amplitude at 1 m/s is higher than at other wind speeds, mainly because the asymmetric multi-edge layout intensifies flow separation instability even at low wind speeds. In Figure 4g (SEH, *N* = 6), the same high amplitude at 1 m/s is observed as for *N* = 5, but at 6 m/s the amplitude is lower than that of *N* = 5. This is because the six-prism distribution is more symmetric, which weakens the vibration response. In Figure 4h (SEH, *N* = 7), high amplitude is maintained over the entire wind speed range, and the lift coefficient amplitude is higher than that of all other SEH configurations and the CEH, indicating that the seven-prism configuration intensifies flow separation instability. In Figure 4i (SEH, *N* = 8), although this configuration has the largest number of prisms, its lift coefficient amplitude is lower than that of *N* = 7 under all wind speeds. This is because eight prisms are nearly continuously distributed around the circumference, making the outer contour approach a nearly circular polygon; the fixing effect on the flow separation points is weakened, thereby reducing aerodynamic instability.

**Figure 5:**
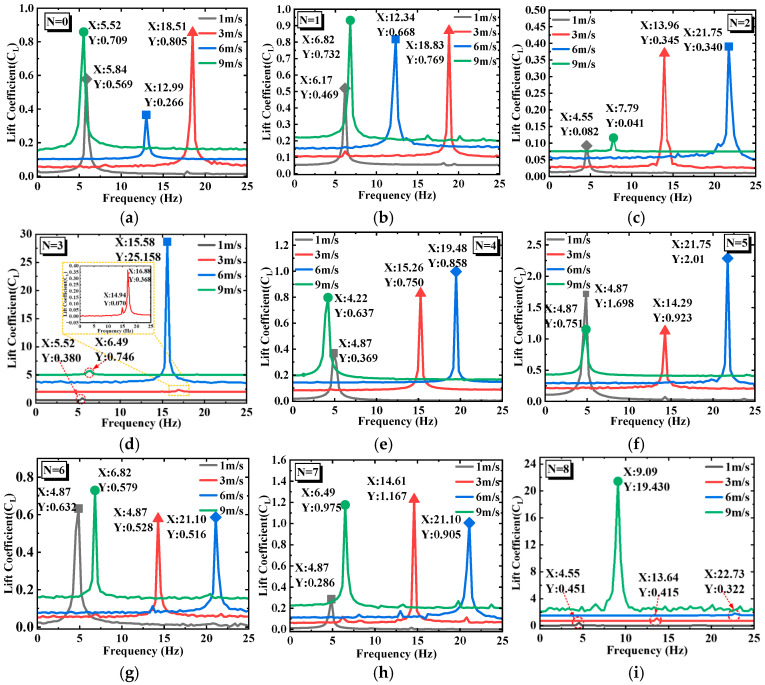
Frequency spectra of the lift coefficients for SEH and CEH under different wind speeds. (**a**) *N* = 0 (**b**) *N* = 1 (**c**) *N* = 2 (**d**) *N* = 3 (**e**) *N* = 4 (**f**) *N* = 5 (**g**) *N* = 6 (**h**) *N* = 7 (**i**) *N* = 8.

Figure 5 presents the frequency spectra obtained by fast Fourier transform (FFT) of the lift coefficient, reflecting the vortex shedding frequency and its energy distribution in the wake of the bluff body under different wind speeds. In general, a clear dominant frequency component exists for all configurations, but the variation in the dominant frequency with wind speed and the number of prisms *N* reveals different fluid–structure interaction mechanisms. In Figure 5a (CEH, *N* = 0), at 1 m/s the dominant frequency is 5.84 Hz with an amplitude of 0.569. When the wind speed increases to 3 m/s, the dominant frequency jumps to 18.51 Hz with an amplitude of 0.805, and the spectrum exhibits a sharp single peak. At 6 m/s, the dominant frequency drops to 12.99 Hz, and at 9 m/s it further decreases to 5.52 Hz, close to the shedding frequency at 1 m/s. In Figure 5b (SEH, *N* = 1), the variation in the dominant frequency is similar to that of the CEH, but at 6 m/s the amplitude is 0.668, higher than that of the CEH at the same wind speed. This suggests that a single prism slightly enhances the vortex shedding intensity but does not alter the VIV-dominated mechanism. In Figure 5c (SEH, *N* = 2), sharp single peaks appear at both 3 m/s and 6 m/s, but the amplitude at 6 m/s is slightly lower and accompanied by high-frequency spikes. This indicates that the two-prism configuration begins to introduce secondary frequency components into the wake, although the dominant frequency remains locked by VIV. In Figure 5d (SEH, *N* = 3), two close dominant peaks (16.88 Hz and 14.94 Hz) appear at 3 m/s, which is the frequency-domain manifestation of the beating phenomenon observed in the time domain. At 6 m/s, a dominant peak at 15.58 Hz appears with an amplitude of 25.158. In Figure 5e (SEH, *N* = 4), the dominant frequencies at 1 m/s and 9 m/s are similar to those of the CEH, with amplitudes slightly lower than those of the CEH, indicating that the “blunting” effect of the four prisms suppresses vibration. In Figure 5f, it can be observed that the dominant frequency distribution of SEH (*N* = 5) under different wind speeds is similar to that of SEH (*N* = 4). In Figure 5g (SEH, *N* = 6), the overall spectral energy density is lower than that of the SEH with *N* = 5, suggesting that the more symmetric six-prism layout weakens the aerodynamic instability and reduces the galloping intensity. In Figure 5h (SEH, *N* = 7), the spectral energy is distributed over multiple frequency components over the entire wind speed range, which is the ideal characteristic for broadband energy harvesting. In Figure 5i (SEH, *N* = 8), the spectral behavior is similar to that of *N* = 3: a dominant peak appears at 9.09 Hz with an amplitude of 19.430 at 9 m/s. However, the overall energy distribution is more concentrated than that of *N* = 7, indicating that an excessive number of prisms makes the flow separation points more uniform, thereby weakening the broadband characteristics.

## 5. Experimental Prototype and Setup

**Figure 6:**
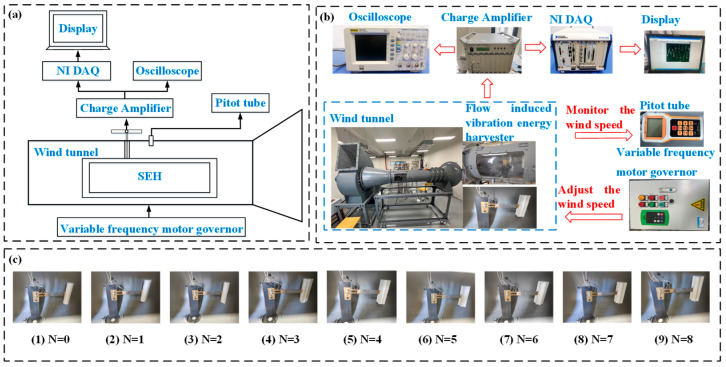
Schematic of the wind tunnel experimental setup and the SEH prototype: (**a**,**b**) Experimental setup, (**c**) The SEH prototype.

The wind tunnel experimental setup is shown in Figure 6a,b. The system consists of a wind tunnel, the SEH, an NI DAQ system, an oscilloscope, a charge amplifier, a Pitot tube, a fan, and a frequency modulator. The SEH is mounted on a support inside the tunnel, with a fan installed in its rear section. Driven by the frequency modulator, the fan adjusts the wind speed via frequency control. The generated airflow passes through a honeycomb flow straightener to produce a stable flow. Under this steady flow, the SEH generates alternating charges, which are amplified by the charge amplifier and then acquired, displayed, and recorded as output voltage by the NI DAQ system. Figure 6c presents the experimental prototype of the SEH. Configuration (1) shows the CEH (*N* = 0) prototype, which serves as the control group for comparison with the SEH. The material and structural parameters of SEH are presented in Table 1. During the electrical performance tests, a fixed load resistance *R_L_* = 100 kΩ was connected to the output of the energy harvester.

## 6. Results and Discussion

**Figure 7:**
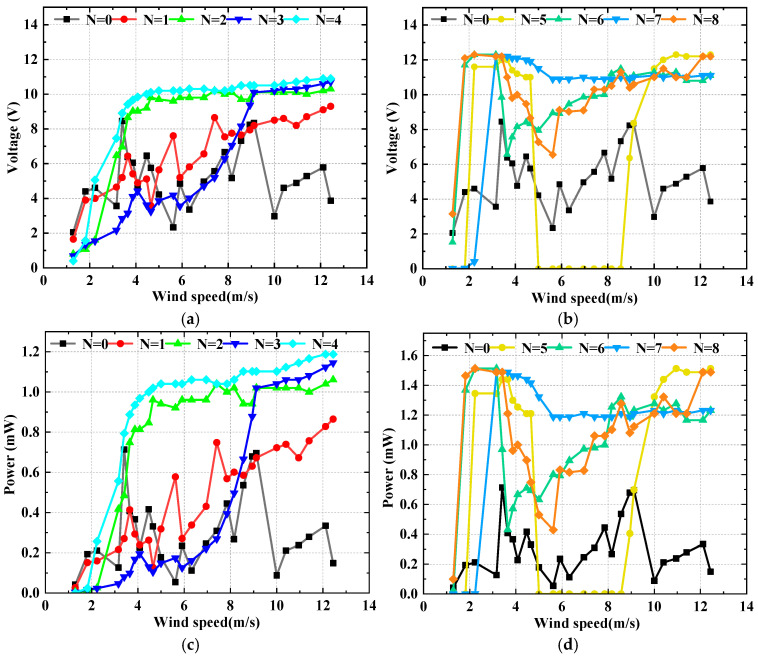
Performance of the SEH and CEH at different wind speeds: (**a**) Output voltage for *N* = *i* (1 ≤ *i* ≤ 4) and CEH; (**b**) Output voltage for *N* = *i* (5 ≤ *i* ≤ 8) and CEH; (**c**) Output power for *N* = *i* (1 ≤ *i* ≤ 4) and CEH; (**d**) Output power for *N* = *i* (5 ≤ *i* ≤ 8) and CEH.

To evaluate the harvesting performance of the SEH, wind tunnel experiments were conducted and the results were compared with those of the CEH (*N* = 0). Figure 7 shows the harvesting performance of the SEH with the number of triangular prisms *N* = *i* (1 ≤ *i* ≤ 8) and the CEH under different wind speeds. Figure 7a,b present the output voltage as a function of wind speed, while Figure 7c,d show the corresponding output power. Overall, the SEH exhibits distinctly better harvesting performance than the CEH. The output voltage of the CEH first increases with wind speed, reaching a first peak of 8.45 V at 3.41 m/s, then decreases, and gradually rises again after 5.62 m/s to a second peak of 8.34 V at 9.12 m/s, with noticeable voltage fluctuations throughout. Correspondingly, the output power of the CEH is relatively high in two narrow bands (3.16~4.65 m/s and 8.16~10 m/s), with a maximum power of 0.714 mW (at 3.41 m/s). In contrast, the SEH with *N* = 7 achieves a maximum output voltage of 12.2 V at 3.41 m/s, corresponding to a power of 1.488 mW, and its effective wind speed range (defined as the continuous interval where the output voltage is no less than 50% of its peak) is broadened to 2.5~12.44 m/s. Compared with the CEH, the SEH (*N* = 7) increases the maximum output voltage by 44.38%, the maximum output power by 108.4%, and expands the effective wind speed range by 198.50%. The significant performance improvement of the SEH is attributed to the introduction of an asymmetric multi-edge geometric perturbation via the circumferentially arranged triangular prisms, which successfully excites the coupled VIV-galloping mechanism.

Further examination of the performance under different *N* values reveals that as *N* increases from 1 to 4, the maximum output voltages of the SEH are 9.3 V, 10.3 V, 10.7 V, and 10.9 V, respectively, with corresponding maximum powers of 0.865 mW, 1.061 mW, 1.145 mW, and 1.188 mW. It can be seen that the overall output performance of SEH (*N* ≤ 4) is not significantly different from that of the CEH. This is because the low-density arrangement of prisms causes only a weak geometric perturbation to the flow field around the cylindrical base, leading to only a moderate improvement in the output performance of SEH (*N* ≤ 4). When *N* = 5, the SEH output voltage sharply rises to 12.3 V (power 1.513 mW) in the low wind speed range (approximately 2~4.5 m/s), but drops sharply to nearly zero in the range of 5~8.56 m/s, and then recovers again above 8.94 m/s. The drop to zero in the 5–8.56 m/s range is caused by a mismatch between the natural frequency of the SEH (*N* = 5) structure and the vortex shedding frequency, leading to chaotic flow and cessation of vibration. For *N* = 6 and *N* = 8, the output voltage (and power) shows a trend of “sharp increase—decrease—then steady increase” with wind speed, indicating some degree of vibration suppression. In contrast, the *N* = 7 configuration maintains continuous, high-level power output over the entire wind speed range, confirming its suitability as the optimal parameter combination.

**Figure 8:**
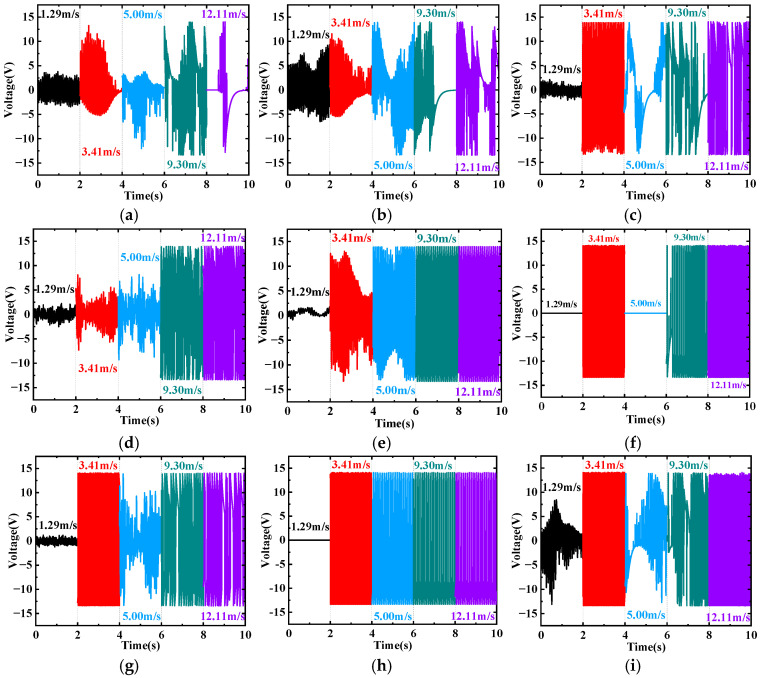
Time histories of the induced voltage for the SEH and CEH at wind speeds of 1.29 m/s, 3.41 m/s, 9.30 m/s, and 12.11 m/s. (**a**) *N* = 0 (**b**) *N* = 1 (**c**) *N* = 2 (**d**) *N* = 3 (**e**) *N* = 4 (**f**) *N* = 5 (**g**) *N* = 6 (**h**) *N* = 7 (**i**) *N* = 8.

Figure 8 shows the time histories of the output voltage for the SEH and CEH at four wind speeds: 1.29 m/s, 3.41 m/s, 9.30 m/s, and 12.11 m/s. In general, the output voltage of all configurations exhibits an approximately sinusoidal waveform, but the amplitude stability and waveform regularity depend significantly on the number of prisms *N* and the wind speed. The output voltage amplitudes of the CEH (*N* = 0) and SEH (*N* = 1) fluctuate considerably and vary nonlinearly with wind speed (Figure 8a,b). For SEH (*N* = 2), resonance occurs at 3.41 m/s, showing a regular periodic waveform. When the wind speed increases to 5 m/s, the vortex shedding frequency deviates from the natural frequency, resonance disappears, and the signal becomes aperiodic. At 12.11 m/s, the non-periodic components weaken, galloping gradually becomes dominant, and the amplitude recovers (Figure 8c). The output voltage of SEH (*N* = 3) increases monotonically with wind speed and stabilizes after exceeding 9.30 m/s (Figure 8d). SEH (*N* = 4) behaves similarly to N = 3, with the output voltage increasing monotonically with wind speed (Figure 8e). For SEH (*N* = 5), the output voltage is near zero at 1.29 m/s, jumps sharply to 12.2 V at 3.41 m/s, but drops abruptly to zero again at 5 m/s, only recovering above 9.30 m/s (Figure 8f). This is due to a severe mismatch between the natural frequency and the vortex shedding frequency, leading to a temporary cessation of vibration. SEH (*N* = 7) exhibits excellent harvesting performance at all selected wind speeds: after exceeding 3.41 m/s, the output voltage remains stable above 12 V, with regular waveforms and high amplitudes (Figure 8h). The *N* = 7 configuration maintains optimal performance over the entire wind speed range. SEH (*N* = 6) and SEH (*N* = 8) also exhibit irregular vibrations at 5 m/s (Figure 8g,i), indicating a certain degree of vibration suppression.

## 7. Conclusions

By systematically optimizing the key geometric parameters—the number of triangular prisms *N* and the circumferential installation angle *α*—this paper proposes a parametrically adjustable star-shaped energy harvester (SEH). The SEH features a cylindrical base with a tunable number of triangular prisms uniformly distributed along its circumference, enabling flexible tailoring of the bluff body aerodynamics. Through conceptual design, theoretical modeling, three-dimensional fluid–structure interaction simulations, and wind tunnel experiments, the regulatory effects of *N* and *α* on the VIV-galloping coupling response and energy harvesting performance are revealed. The main findings are as follows:

(1) Significant enhancement of lift characteristics: Simulation results demonstrate that *N* plays a key role in regulating the lift force acting on the structure. Under the optimal parameter combination (*N* = 7, *α* = 51.4°), the maximum lift force of the SEH reaches 0.244 N/m at a wind speed of 9 m/s, which is 225% higher than that of the CEH (0.075 N/m). This performance improvement is attributed to the asymmetric multi-edge layout that intensifies flow separation instability and increases the amplitude of the fluid excitation force.
(2) Parametric optimization of energy harvesting performance: Experimental validation shows that under the same optimal parameter combination (*N* = 7, *α* = 51.4°), the SEH increases the maximum output voltage by 44.38% and the maximum output power by 108.4% compared with the CEH. The effective wind speed range (defined as the continuous interval where the output voltage is no less than 50% of its peak) is broadened from the narrow bands of the CEH (3.16~4.65 m/s and 8.16~10 m/s) to 2.5~12.44 m/s, corresponding to an expansion of 198.50%. The essence of this performance leap lies in the successful excitation of a smooth transition and synergistic coupling between VIV and galloping over the entire wind speed range through the optimization of *N* and *α*.

Through systematic optimization of the key geometric parameters (prism number and circumferential angle), this study achieves synergistic regulation of flow-induced vibration modes and performance enhancement, providing a quantitative basis for the parametric design of efficient low-speed wind energy harvesters. Future work will further introduce artificial intelligence tools to conduct multi-objective optimization studies on additional geometric parameters, including the incoming flow angle, the aspect ratio of the triangular prisms, and the spacing between the prisms and the central cylinder.

## Figures and Tables

**Table 1:** Material and structural parameters of the SEH.

Parameter	Material	Size
circular bluff body	plastic foam	*D* = 32 mm, *H* = 120 mm
triangular prism	plastic foam	*a* = 11 mm, *b* = 11 mm, *H* = 120 mm
beam	copper	180 mm × 20 mm × 1 mm
piezoelectric patch	PZT-5H	24 mm × 20 mm × 1 mm

## Data Availability

The original contributions presented in this study are included in the article. Further inquiries can be directed to the corresponding author.
